# Is an association of acro-osteolysis, bone fragility, and enchondromatosis a newfound disease caused by an amplification of PTHLH? A case report

**DOI:** 10.1186/s12969-022-00720-8

**Published:** 2022-07-30

**Authors:** Stéphane Echaubard, Céline Pebrel-Richard, Aurélie Chausset, Jean-Louis Kemeny, Etienne Merlin, Fanny Laffargue

**Affiliations:** 1 Service de Pédiatrie, CHU de Clermont-Ferrand, CHU Estaing, 1 place Lucie & Raymond Aubrac, 63003 Clermont-Ferrand, France; 2 Service de Cytogénétique Médicale, CHU de Clermont-Ferrand, 63003 Clermont-Ferrand, France; 3 Unité CRECHE, INSERM CIC 1405, CHU de Clermont-Ferrand, 63003 Clermont-Ferrand, France; 4 Service d’Anatomo-Pathologie, CHU de Clermont-Ferrand, 63003 Clermont-Ferrand, France; 5 Service de Génétique Médicale, CHU de Clermont-Ferrand, 63003 Clermont-Ferrand, France

**Keywords:** Acro-osteolysis, *PTHLH* gene, Rare disease, 12p11.22p11.23 duplication

## Abstract

**Background:**

Acro-osteolysis (AO) refers to resorption of the distal finger and toe phalanges. It displays two patterns: (i) diffuse AO and (ii) transverse or bandlike AO. AO can be a sign of local distress (e.g. of toxic origin), but is very often a sign of a constitutional or systemic acquired disorder.

**Case presentation:**

A 15-year-old girl was referred to a paediatric rheumatologist for recurrent pain in her fingertips. She presented a particular cross-sectional AO associated with the presence of intraosseous cysts and bone fragility with atypical fractures. Initial laboratory tests and radiological examination did not allow an etiological diagnosis. Genetic studies revealed a 12p11.22-p11.23 microduplication of 900 kb including the *PTHLH* (parathyroid hormone-like hormone) gene, which encodes for a hormone involved in the regulation of endochondral ossification and differentiation of chondrocytes, via its PTHLH receptor.

**Conclusions:**

To date, 12p11.22-p11.23 duplications have been reported in five families with skeletal abnormalities, and in particular AO and enchondromatosis associated with bone fragility. This new observation, added to the other reported cases, suggests a close relationship between the presence of this microduplication and the skeletal abnormalities found in the patient. We suggest the descriptive name ABES (acro-osteolysis, bone fragility and enchondromatosis syndrome) to designate this disorder.

**Supplementary Information:**

The online version contains supplementary material available at 10.1186/s12969-022-00720-8.

## Background

Acro-osteolysis (AO) is a clinical symptom and a radiological sign characterized by the destruction of the distal phalanx of the fingers and toes. Two types are described: (i) resorption of the distal end of the phalanx, and (ii) damage to the body of the phalanx (band or transverse AO). The second type is characterized by the presence of a hypodense line through the body of the distal phalanx by standard radiography.

Clinically, AO presents initially as a “drumstick” deformation of the fingers. As the bone breaks up, the deformation gradually worsens, giving the fingers and toes a shortened, soft, enlarged appearance. Deformations are sometimes associated with other clinical signs (short stature, deafness, skin abnormalities, neuropathy, etc.) related to the etiology.

There are many causes of AO, both constitutional and acquired [1]. Acquired AO is usually asymmetrical and may affect only one phalanx. Environmental causes are dominated by repetitive microtrauma resulting only in affected fingers. Toxic substances such as vinyl chloride or snake venom can cause more diffuse forms but not symmetrical damage. Phalangeal tumours are singly located and associated with typical radiological abnormalities.

In the context of non-constitutional general diseases, acquired AO is only an associated sign, in most cases secondary to the original disease. The cause may be neurological (diabetic neuropathy), vascular (systemic lupus erythematosus, scleroderma, vasculitis with poly-angiitis), inflammatory (psoriasis, psoriatic rheumatism) or infectious (e.g. leprosy).

Hyperparathyroidism can also lead to AO, usually more diffuse and generally associated with a broader context of bone demineralization, which may speciously suggest a genetic origin. Phosphocalcic exploration is also essential for establishing one AO etiology.

Constitutional forms are unusual, but recognized (e.g. in some lysosomal diseases). These diseases are very rare and associate AO with other clinical signs. These signs guide diagnosis: they can be bone manifestations (fragility, deformities, malformations, worm-like bones), skin signs (pachydermia, hyperhydrosis, lipodystrophy), neurological signs (loss of sensitivity, neuropathy, muscle weakness) or haematological signs [2–15]. The best known is the Hajdu-Cheney syndrome linked to a *NOTCH2* mutation [16, 17]. Although these syndromes are numerous, they affect only a few patients. None of them associates AO and multiple enchondromas. A summary of clinical and genetic features is given in Table 1.

**Table 1:** A summary of the characteristics of genetic acro-osteolysis

	Pycnodysostosis	Neuropathy hereditary sensory and autonomic type iia / iib = neurogenic acroosteolysis	Pachydermoperiostosis	Haim-munk syndrome	Warburg-cinotti syndrome	Mandibulo-acral dysplasia with type a/type b lipodystrophy	Penttinen syndrome	Hajdu-cheney syndrome	*PTHLH* amplification Acroosteolysis Bone fragility and Echondromatosis Syndrome
OMIM	265,800	201,300	259,100	245,010	618,175	248,370 608,612	601,812	102,500	
Pain			X					X	X
Mandibular lesions						X	X	X	Once (case)
Long bones involvement	X		X			X	X	X	X
Rachis involvement	X					X	X	X	
Other bone abnormalities	X		X	X	X	X	X	X	X
Bone fragility	X	X	X					X	X
Skin, nails, and hair involvement	X	X	X	X	X	X	X	X	
Sensory impairment		X							Deafness?
Central nervous system impairment								X	
Neurosensory disorder					X		X	X	
Dysmorphia	X		X	X	X	X	X	X	
Dental anomalies	X		X	X	X	X	X	X	
Visceral involvement			X		X	X		X	
Growth retardation or short stature	X					X		X	
Increased stature			X				X		
Gene	*CTSK*	*WNK1* *FAM134B*	*HPGD*	*CTSC*	*DDR2*	*LMNA* *ZMPSTE24*	*PDGFRB*	*NOTCH2*	*PTHLH*
Chromosome	1q21	12p13.33 5p15.1	4q33-q34	11q14.2	1q23.3	1q22 1p34.2	5q32	1p13-p11	12p11.22-p11.23
Inheritance	Autosomal recessive	Autosomal recessive	Autosomal recessive	Autosomal recessive	Autosomal dominant	Autosomal recessive	Autosomal dominant	Autosomal dominant	?

Here we report on a female patient aged 15 years with AO who presented deformation of the fingers, pain, and a particular clinical and radiological aspect, related to a microduplication of 900 kb at 12p11.22-p11.23 including the *PTHLH* (parathyroid hormone-like hormone) gene.

## Case presentation

A 15-year-old girl of Caucasian French origin was referred to a paediatric rheumatologist for recurrent pain in her fingertips in the last few months, with gradual onset. She had no past medical history, and no surgical history except for one tonsillectomy. She was born with a monozygous twin to non-consanguineous parents.

At first visit, she complained of pain in her fingers and toes that had appeared a few months previously. Progressively, she presented drumstick fingers, and then multiple bone pain localization (metatarsals, wrists).

Pulmonary function test, cardiac investigation and capillaroscopy were normal. Sensory or motor impairment, joint or skin involvement, calcinosis, Raynaud’s syndrome, signs of dysthyroidism or diabetes were not found. Respiratory examination was normal, and the differential diagnosis of hypertrophying osteoarthropathy could be excluded.

otological examination found an auditory defect at 45 dB only at 2000 Hz. Ophthalmological examination reported mild hyperopia.

She presented some clinical features not reported in other members of her family: slender frame, high forehead, facial asymmetry, moderate scoliosis. Her growth was regular and homogeneous.

The patient declared no repeated microtrauma of extremities or any unusual exposure to vinyl chloride.

### Laboratory tests

Initial laboratory tests found no inflammatory syndrome, and blood count was normal. There were no autoantibodies or immune deficiency. Phosphocalcic exploration was considered normal. The corrected value of calcaemia was a little low (2.02 mmol/L) but with a normal level of ionized calcium. This could be explained by a level of vitamin D3 below standard values but could not explain the symptoms. Leprosy was not looked for, given no travel in affected areas and a perfectly normal lifestyle and hygiene.

### Radiological examination

**Fig. 1:**
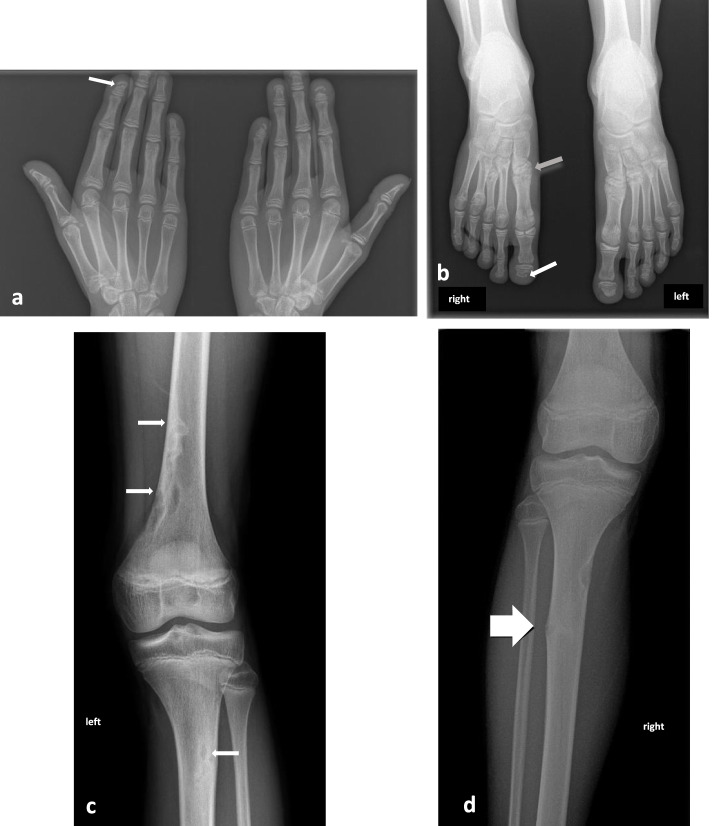
Radiological examination at first presentation. **a** Presence of hyperclear, transverse bands on the distal phalanges, characteristic aspect of transverse acro-osteolysis (white arrow). **b** Presence of transverse lysis of the phalanges (white arrow) and modification of the bone structure of the metatarsals, appearance of a “fracture callus” (grey arrow). The overall symmetrical condition is noteworthy. **c** Numerous ovoid lesions, with clear centre and hyperdense outline reminiscent of intraosseous chondromes (white arrows). There is no sign of malignancy. **d** Appearance of a small spur with bellowing of the cortex, probably corresponding to a cortical point fracture (white arrow) and presence of chondromes

The first radiological examination found transverse AO of all the distal phalanges in both fingers and toes (Fig. 1a and b) and confirmed scoliosis.

In some metatarsals, the bone was found to have a nodular, heterogeneous appearance, suggestive of a fracture-consolidation cycle.

A number of enchondromas and cortical defects were also found, located on the metaphysical part of the fibula and tibia (Fig. 1c and d). Sclerosis was found in the left horizontal branch of the mandibula, with no malignancy.

Since the symptom of AO was retained, a diagnostic evaluation was carried out.

### Additional search

Besides medical examination, a bibliographical search of the MEDLINE biomedical database was made using the keywords “acro-osteolysis” and “enchondromatosis” to identify any previously reported cases.

The clinical and radiographic examination initially directed diagnosis towards a Hajdu-Cheney syndrome, soon discarded owing to the presence of long-bone lesions and the absence of *NOTCH2* mutation. Despite the absence of obvious deafness and dental anomalies, the diagnosis of familial osteolysis related to mutations in the *TFNRSF11A* gene was also suspected. The molecular study of this gene did not reveal any abnormality. At the same time, the laboratory test results ruled out acquired or lysosomal AO. In view of the atypicality of this AO, genetic investigation on DNA microarray was prescribed. Patient’s and legal guardian’s consent were obtained prior to genetic testing.

### Histology

A bone biopsy was performed during the femoral graft intervention. It showed viable bone span, surrounded by new bone with an irregular contour area in some cases, associated with a resorption area sometimes filled with osteoclasts or osteoid spans, surrounded by osteoblasts and fibrosis. These results suggest a hyperactivity of osteoclasts, with bone degenerative involvement and fibrosis, which can explain fragility and fractures (Additional file 1). These kinds of anomaly had already been noted in a previous case [18].

### Genetic investigation

#### Microarray-based comparative genomic hybridization (array CGH)

Comparative genomic hybridization (CGH) array analysis was performed on genomic DNA isolated from peripheral blood lymphocytes (Maxwell 16 Promega© kit Low Elution Volume) using an oligonucleotide microarray (60 K; Agilent Technologies, Palo Alto, California) according to the manufacturer’s protocol to identify possible genomic copy number aberrations. The results were interpreted with the Agilent Cytogenomics 4.03.12 software. Genomic positions were defined using NCBI37/hg19.

#### Quantitative multiplex fluorescence - PCR (QMF-PCR)

QMF-PCR was performed as previously described by Niel et al. [19] on a Professional thermocycler (Biometra) to confirm the aberration identified by array-CGH. Primers (sequences available on request) were designed in *PTHLH* for exons 1, 2 and 3. Exon 4 of *CFTR* [cystic fibrosis transmembrane conductance regulator on chromosome 7] and exon 6 of *PLP1* [proteolipid protein 1 on chromosome X] genes were co-amplified as controls. Briefly, the principle is based on comparisons of the fluorescent profiles of multiplex PCR fragments, the amplification being stopped at the exponential phase. This procedure allows the detection of heterozygous deletions (twofold reduction of fluorescence intensity) and heterozygous duplications (1.5-fold increase). The forward primers were labelled with the fluorescent phosphoramidite 6-FAM dye. The PCR reactions were performed in duplicate in 25 µl reactions using the QIAGEN Multiplex PCR kit (Qiagen, Courtaboeuf, France), with 300 ng of genomic DNA and a mix of primers (concentration range 0.1–0.8 mM). The reaction started with an initial denaturation of 15 min at 95˚C, followed by 19 cycles at 95˚C for 30 s, 55˚C for 30 s, and 72˚C for 45 s, and a final extension of 10 min at 72˚C. Then 2 ml of the purified PCR products were added to 9.8 ml formamide and 0.2 ml Genescan-500 Rox size standard (Applied Biosystems, Foster City, CA, USA). The fluorescent PCR products were separated on a 16-capillary sequencer (ABI PRISM 3100 Genetic Analyzer, Applied Biosystems). The results were processed by Genescan 3.7 software (Applied Biosystems) to obtain electropherograms for each sample. Each product was identified by its size, and fluorescence intensities were correlated to the copy number of the relevant exons.

**Fig. 2:**
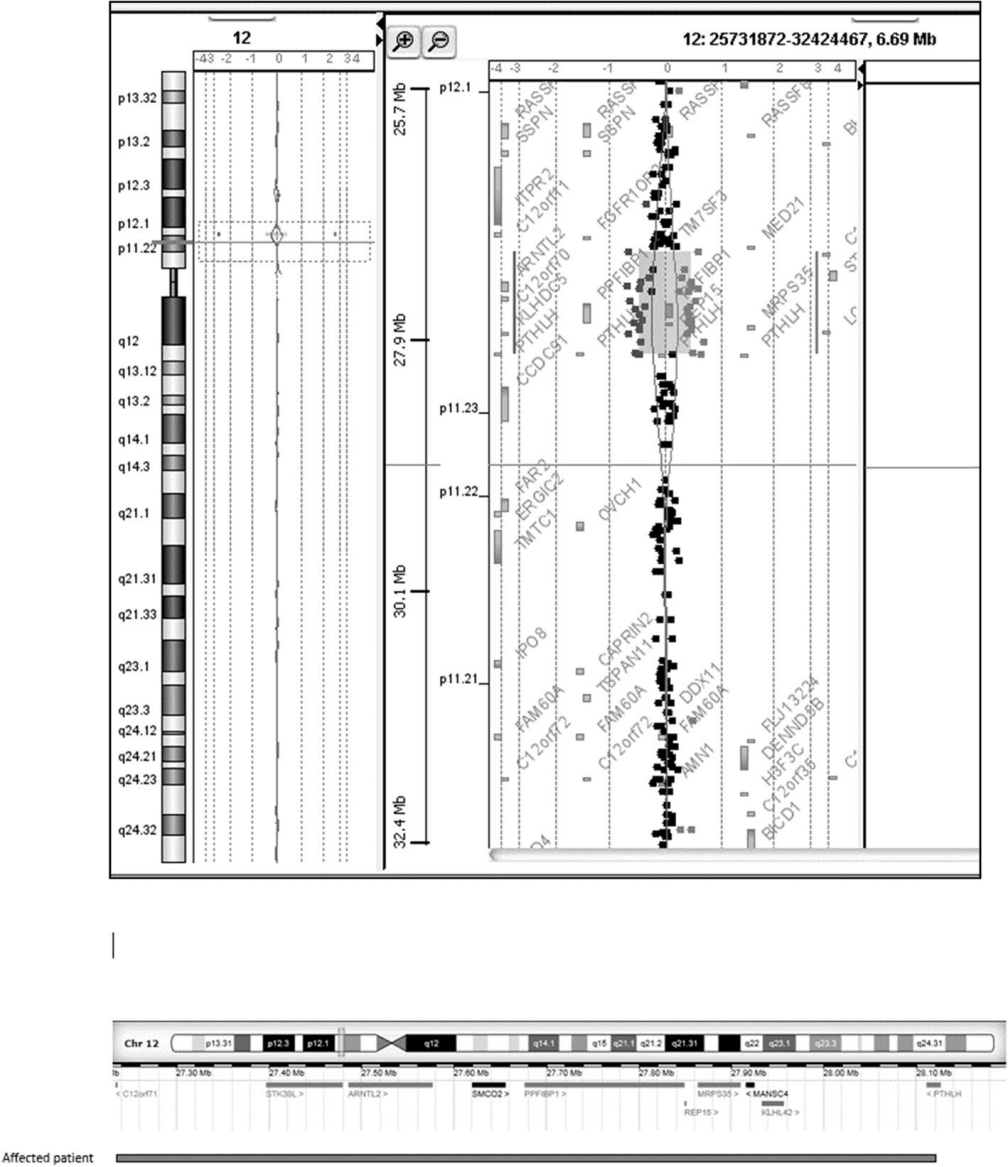
Chromosomal analysis on DNA microarray. An amplification of about 900 kb at the short arm of a chromosome 12 (12p11.23p11.22) between genomic positions 27,232,251 − 28,123,884 (NCBI Build37) was identified and confirmed by QMF-PCR (inherited from mother)

Array CGH revealed a gain of genomic copy number at chromosome 12p11.22-p11.23 with a size of 900 kb encompassing the *PTHLH* gene (Fig. 2) and confirmed by QMF-PCR. According to the February 2009 human reference sequence (NCBI Build 37-Hg19) on the University of California Santa Cruz (UCSC) Genome Browser, the duplication occurred in chromosome 12, extending from base 27,232,251 (the first-deleted probe) to base 28,123,884 (the last-deleted probe). No other abnormality was observed, apart from well-known benign copy number variations. Follow-up kin study showed an amplification of *PTHLH* in the proband’s mother (data not shown). In French law, genetic examinations can be prescribed for a minor only if he or she can personally benefit from immediate preventive or curative measures, which was not the case here insofar as the patient’s sister, a minor, had no bone symptoms.

### Clinical evolution

**Fig. 3:**
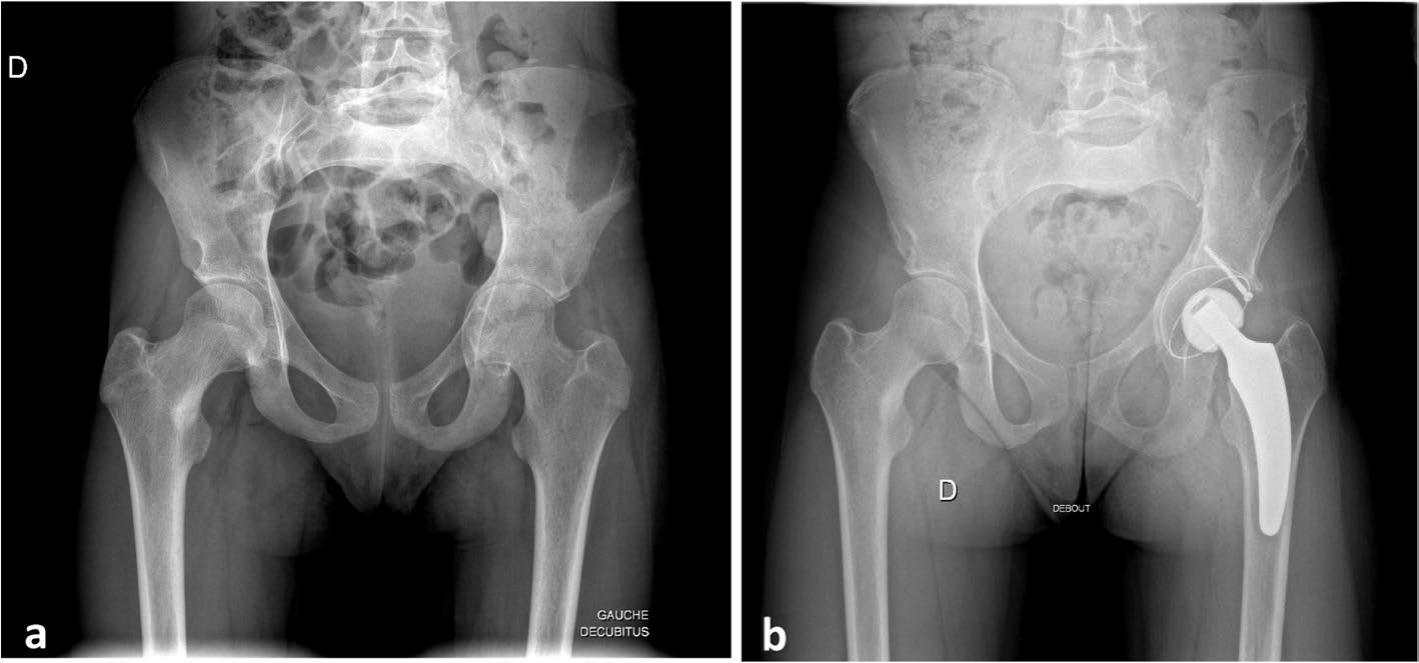
Pre-surgical (**a**) and post-surgical (**b**) evolution of the cotyloid fracture. Marked acetabular pelvic protrusion indicates total prosthesis. Note the left iliac defect corresponding to the graft

Because of reported cases of repeated fractures in the literature, we started a bisphosphonate treatment (9 mg/kg/year, 3 sequences of 3 days, every four months). Recurrent pain (first mechanical then mixed) was treated with self-administered analgesics after a period of therapeutic education, resulting in overall pain relief in the extremities. Two years after the first visit, the patient presented a cotyloid fracture initially treated with iliac grafting. Unfortunately, the hip evolved towards destruction of the cotyloid bottom, requiring total hip prosthesis at age 17 years (Fig. 3). Shortly after surgery, a severe L5-S1 spondylolisthesis appeared with posterior sacrum rocking requiring anterior-posterior arthrodesis (Additional file 2). Since then, the patient has regularly presented atypical mild fractures: long bones and ribs requiring brief immobilization. These may occur without trauma but induced persistent pain and reduced quality of life. The last serious fracture was femoral, treated by bone graft, unfortunately with failure of the treatment. Bisphosphonates are regularly adjusted according to the progress of the bone remodelling process (alkaline phosphatase, beta-crosslaps, procollagen type 1 N-terminal propeptide). We did not use denosumab as the patient presented lesions of the mandibulae with a risk of osteonecrosis. We cannot be sure that this treatment is effective in this case, but there are no alternatives, and no perspective of good progression at this point.

## Discussion and conclusion

AO is a radiological and clinical manifestation of an underlying disease that may or may not be of constitutional origin.

Here we describe the case of a 15-year-old girl who presented an AO with deformation of the fingers, pain, and specific bone abnormalities. Initial assessment did not allow an etiological diagnosis. However, a cytogenetic study revealed an interstitial microduplication of 900 kb at 12p11.22-p11.23. The duplicated region had 10 Refseq genes including the *PTHLH* gene.

This duplication partially overlapped a polymorphism reported in the database of genomics variants, hindering interpretation. Besides this polymorphism, five genes remain: *REP15*, *MRPS35*, *MANSC4*, *KLHDC* and the *PTHLH* gene. To date, in the literature, duplications 12p11.22-p11.23 have been described in five families. The first patient described by Collinson et al. presented short stature and very unusual generalized symmetrical enchondromatosis of the tubular bones [20]. Three patients described by Gray et al. presented AO, cortical irregularity of long bones and metadiaphyseal enchondromatosis [18]. Recently Limenis et al. published a review on differential diagnosis of AO and its distinguishing features, with particular attention paid to types more commonly encountered in paediatrics [21]. Our article reports new findings on the relationship between this amplification and this disease expressed by an AO and an anomaly of the long bones. It also provides data on anatomopathology and clinical evolution. Flöttmann et al. reported on a three-generation pedigree with short humerus, curved radius, and a specific type of severe brachydactyly with features of types E and A1 but without the enchondromatosis and AO hitherto described [22]. Finally, Tacke et al. reported on a patient with multiple skeletal abnormalities including chondrodysplasia, lesions radiographically resembling enchondromas and posterior rib deformities leading to a severe chest deformity [23]. No AO was observed in this patient (probably not yet present owing to the young age of the patient compared to the other observations).

A summary of the phenotypic characteristics of our patient and those described in the literature is given in Table 2.

**Table 2:** Phenotypic characteristics of our patient and those described in the literature

			Collinson et al., 2010 [20]	Gray et al., 2014 [18]	Gray et al., 2014 [18]	Gray et al., 2014 [18]	Flottmann et al., 2016 [22]	Flottmann et al., 2016 [22]	Flottmann et al., 2016 [22]	Tacke et al., 2021 [23]
Patient	**Our patient**	**Our patient’s mother**	P1	P2	P3	P4	P5	P6	P7	P8
					Father of P2			Mother of P5	Sister of P5	
Family	Family 6	Family 6	Family 1	Family 2	Family 2	Family 3	Family 4	Family 4	Family 4	Family 5
Copy number variation on chromosome 12p11.22	900 kb Duplication including *PTHLH*	900 kb Duplication including *PTHLH*	Duplication including *PTHLH*	850 kb Duplication including *PTHLH*	850 kb Duplication including *PTHLH*	500 kb Duplication including *PTHLH*	70 kb Duplication including *PTHLH*	70 kb Duplication including *PTHLH*	70 kb Duplication including *PTHLH*	898 kb Duplication including *PTHLH*
Inheritance	Maternal	Unknown	*De novo*	Paternal	Unknown	*De novo*	Maternal	Maternal	Maternal	*De novo*
Homogene/mosaic	homogene	homogene	homogene	homogene	mosaïc	mosaïc	homogene	homogene	homogene	homogene
Short stature			+			+		+		+
Skeletal anomalies	+	+	+	+	-	+	+	+	+	+
Shortened limbs or phalanges/asymmetric limbs		-	+	+	-	+	+	+		
Bone/finger/joint deformity	+	-	+	+	-		+	+	+	+
Bone lesions	+	-	+	+	-	+	+	-	+	+
	Acro-osteolysis, fracture-consolidation cycle resorption areas and fibrosis, multiple endochromatosis,	No osteolysis	Brachydactyly type E with cone-shaped epiphyses	Acro-osteolysis osteoporosis		Acro-osteolysis, microscopic anomalies of bone formation, radiolucencies	Abnormal ossification and epiphyseal development bones pseudarthrosis		Delayed epiphyseal maturation, delayed carpal ossification	Chondrodyspla-sia, lesions resembling enchondromas fractures
Other	Facial asymmetry, pain	Post-axial polydactyly	Enchondroma -tosis	Coarse facial features, osteoporosis		Scoliosis	Cervical iordosis	Limitation prono -supination		Swelling and limited mobility of right elbow

PTHLH is a humoral factor known to regulate the balance between proliferation and differentiation of chondrocytes during endochondral bone development. It is produced by the perichondrial and chondrocyte cells, which grow during skeletal development, and it maintains these cells in a proliferative state by a paracrine mechanism. It has a large structural homology with parathyroid hormone (PTH). Mutations and deletions of the *PTHLH* or *PTHR1* genes (encoding the PTHLH protein receptor) cause several types of skeletal dysplasia, including brachydactyly type E, Eiken syndrome, Jansen’s metaphysical chondrodysplasia, and Blomstrand’s chondrodysplasia. Given the role of the PTHLH protein, it is likely that a variation in the number of copies of this gene is involved in the patient’s bone disease. Studies in mice showed that overexpression of PTHLH was responsible for a form of dwarfism with short limbs and delayed endochondral ossification [24]. The 12p11.22 replication most recently described by Flöttmann et al. is also the smallest reported to date (about 70 kb) and contains only the *PTHLH* gene [22]. Although it appears to cause skeletal signs comparable to those described in patients described by Gray et al. [18] and Collinson et al. [20] (short humerus, radial abnormalities, brachydactylia), AO associated with bone cysts is not present in patients with this small duplication. Importantly, the duplications described in these two articles, of larger size, respectively 851 kb and 502 kb, in addition to the *PTHLH* gene and besides the polymorphism previously mentioned, contained three additional genes: *REP15*, *MRPS35* and *KLHDC*. *MRPS35* encodes a mitochondrial ribosomal protein. *REP15* is involved in the recycling of the transferrin receptor. The *KLHDC* gene belongs to the Kelch-like gene family, particularly expressed for limbs in mice during the embryonic period. All these data suggest that the isolated duplication of the *PTHLH* gene alone may not suffice to generate an AO process associated with enchondromatosis, but is the likely cause of other skeletal growth abnormalities identified in the literature and consistent with the results of studies in mice. It could be argued that the duplication of genes included in the larger genomic imbalances, such as those described by Gray et al. and Collinson et al., or in our study, could also affect the bone phenotype, modulating the expression of *PTHLH* or exerting a synergistic effect. Finally, it is possible that these duplications 12p11.22-p11.23, including the *PTHLH* gene, reflect redesigns of varying phenotypic expressivity that explain the intra-family and inter-family phenotypic differences. For example, our patient’s mother carried the same amplification but had no clinical or radiological sign of AO or any enchondromatosis. She had no disease or comorbidity, except obesity. One possible explanation for this phenotypic variability is the “multiple hit”, whereby a second insult is needed to produce a more severe clinical phenotype, via an additive or synergistic effect on other skeletal growth pathways. The second hit might be a disruptive single base-pair mutation in a phenotypically related gene or even an environmental event. Concerning the monozygotic twin sister, we could not legally perform a test because she was asymptomatic. This suggests variable penetrance of the disease, or the involvement of regulatory genes. The reproducibility of symptoms to *PTHLH* amplification in the different case reports suggests a causal relationship.

A query of the Database of Chromosomal Imbalance and Phenotype in Humans using Ensemble Resources (DECIPHER; https://decipher.sanger.ac.uk) identified 11 patients, with detailed phenotype descriptions, presenting with a 12p amplification encompassing at least *PTHLH*. Skeletal anomalies were identified in 6/11 patients and features overlapping our patient’s in 3/6 patients with skeletal anomalies (308,811, 385,237 and 295,002). We note that the variation identified overlaps a small duplication, only including the *PTHLH* gene, in patient 308,811 associated with radial bowing, short humerus and type A1 brachydactyly and most likely corresponding to the duplication described by Flöttmann et al. [22].

To date, 12p11.22-p11.23 duplications have been reported in five families with skeletal abnormalities, and in particular AO and enchondromatosis associated with bone fragility. This new observation, associated with the few cases recently reported in the literature, suggests a close relationship between the presence of this microduplication and the skeletal abnormalities found in the patient, so strengthening the hypothesis of a new nosological entity with a close relationship with disruption of the PTHLH-PTHR1 signalling pathway during skeletal development. The clinical features seem to be variable, possibly by regulation sequences included in the duplication area, and more explorations are needed to understand the mechanisms. We suggest the descriptive name ABES (acro-osteolysis, bone fragility and enchondromatosis syndrome) to designate this disorder.

## Supplementary Information

**Supplementary** **Material** **1.** Bone histology performed during the femoral graft intervention. There was viable bone span, surrounded by new bone with an irregular contour area (*), associated with resorption area filled with osteoclasts or osteoid spans, surrounded by osteoblasts and fibrosis (black arrow).

**Supplementary** **Material** **2.** Spondylolysis L5-S1 (black arrow), associated with spondylolisthesis of nearly 50% (white arrow). There is a very marked sacral backslide that requires surgical correction.

## Data Availability

Data are available by request.

## Abbreviations

AO: acro-osteolysis
CGH: comparative genomic hybridization
PTH: parathyroid hormone
PTHLH: parathyroid hormone-like hormone
PTHR1: parathyroid hormone 1 receptor
