# Supplementary figures and images for: Is an association of acro-osteolysis, bone fragility, and enchondromatosis a newfound disease caused by an amplification of PTHLH? A case report
Source: Pediatr Rheumatol Online J. 2022 Jul 30;20:58. doi: 10.1186/s12969-022-00720-8 (PMC9338489; doi:10.1186/s12969-022-00720-8)

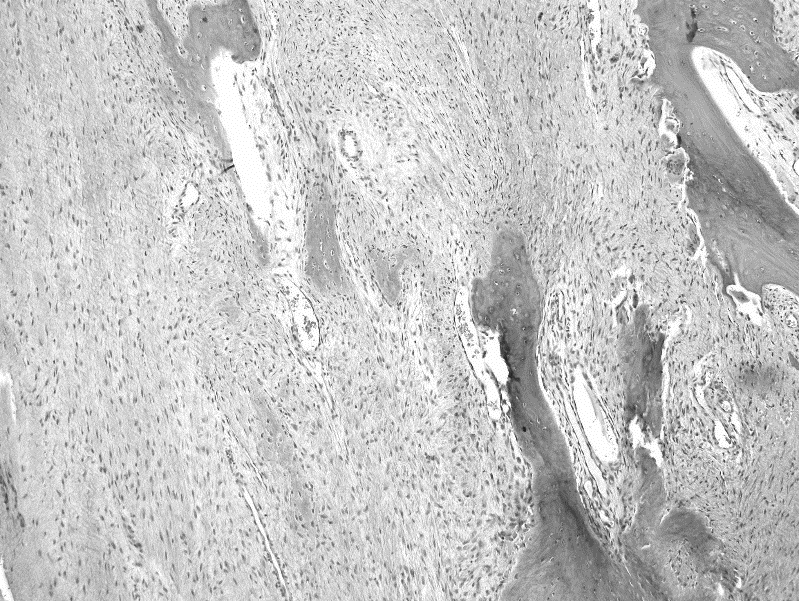

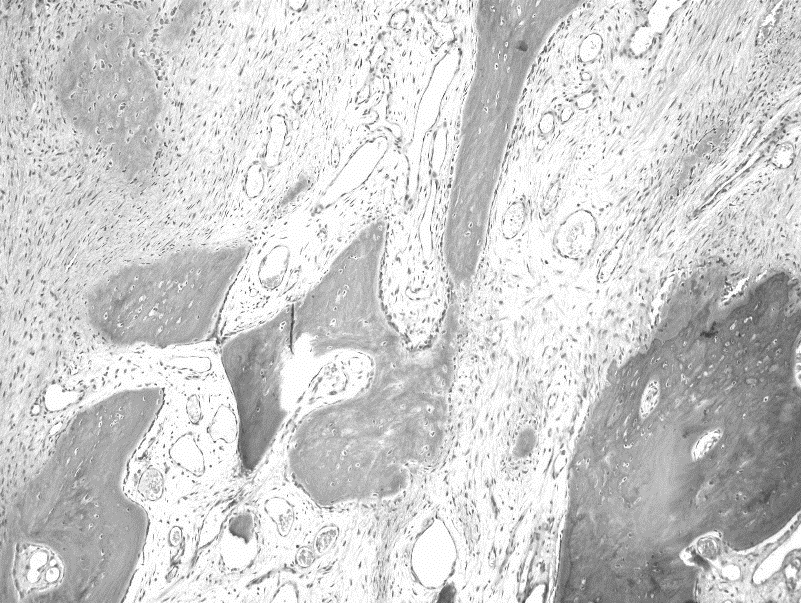


*****

Additional file 1

Supplement: Supplementary file 1 — Supplementary Material 1. Bone histology performed during the femoral graft intervention. There was viable bone span, surrounded by new bone with an irregular contour area (*), associated with resorption area filled with osteoclasts or osteoid spans, surrounded by osteoblasts and fibrosis (black arrow). [file 12969_2022_720_MOESM1_ESM.docx]

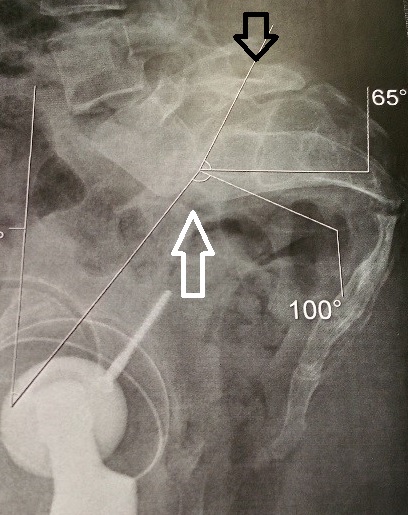


Additional file 2

Supplement: Supplementary file 2 — Supplementary Material 2. Spondylolysis L5-S1 (black arrow), associated with spondylolisthesis of nearly 50% (white arrow). There is a very marked sacral backslide that requires surgical correction. [file 12969_2022_720_MOESM2_ESM.docx]
